# Fast-Tracking of Publication Times of Otolaryngology Papers During the COVID-19 Pandemic

**DOI:** 10.1055/s-0043-1767806

**Published:** 2024-02-05

**Authors:** Irit Duek, Nidal Muhanna, Yahav Oron, Yohai Shraga, Omer J. Ungar

**Affiliations:** 1 Department of Otolaryngology, Head and Neck Surgery and Maxillofacial Surgery, Tel Aviv Sourasky Medical Center, Sackler School of Medicine, Tel-Aviv University, Tel Aviv, Israel

**Keywords:** COVID-19, pandemic, publishing, research, otolaryngology head and neck surgery, ORL-HNS

## Abstract

**Introduction** The outbreak of COVID-19 has produced an unprecedented number of trials and articles.

**Objective** To study the impact of the COVID-19 pandemic on otolaryngology-head and neck surgery (ORL-HNS) journal processing times.

**Methods** Original papers search of published in selected ORL-HNS journals in terms of times from submission-to-acceptance (S-A), acceptance-to-first online publication (A-P), and submission-to-online publication (S-P). Papers were divided into those published in the pre-COVID-19 era and those during the COVID-19 era. The latter were further divided into unrelated to COVID-19 and related to COVID-19.

**Results** A total of 487 articles from 5 selected ORL-HNS journals were included, of which 236 (48.5%) were published during the pre-COVID-19 era and 251 (51.5%) were published during the COVID-19 era. Among them, 180 (37%) papers were not related to COVID-19, and 71 (14.5%) were related to COVID-19. The S-A duration of COVID-19-related articles was significantly shorter compared with that of papers submitted in the pre-COVID-19 era and to papers submitted in the COVID-19 era but unrelated to COVID-19 (median 6 to 34 days compared to 65 to 125 and 46 to 127, respectively) in all 5 journals. The most prominent reductions in S-A and S-P times were documented in the laryngology and otology/neurotology disciplines, respectively.

**Conclusions** Processing times of the included papers were significantly shorter in most of the selected ORL-HNS journals during the COVID-19 era compared with the pre-COVID-19 era. COVID-19-related papers were processed more rapidly than non-COVID-19-related papers. These findings testify to the possibility of markedly expediting S-P times and hopefully set a precedent for postpandemic publishing schedules.

Level Of Evidence: 5

## Introduction

Since 30 January 2020, when COVID-19 was defined by the World Health Organization as a public health emergency of international concern (PHEIC), 1 research regarding many aspects of COVID-19 is being done at an exceptional volume and rate. The outbreak of COVID-19 has produced an unprecedented interest from the medical and nonmedical communities, as well as a remarkable response from healthcare practitioners, the scientific community, and support from biomedical publishers. The scientific community responded to the crisis by extensive mobilization of significant research resources with the aim of shedding light on the virus' characteristics and mechanisms of its transmission, as well as clinical aspects of the disease, prevention, and management strategies. The number of clinical trials on COVID-19 currently exceeds 6,340 registrations at ClinicalTrials.gov. Trial results are being published quickly once data collection is completed. Scientific journals have reacted to both the epidemiological and information crisis in accordance with their role in the transmission of new scientific information – swiftly, effectively, and responsibly. 2

The number of articles arising from clinical studies and observations continues to grow at an unparalleled pace. In January 2020, a sharp growth in the number of COVID-19-related publications was documented in PubMed. Many publishers have waived publication fees, accelerated review processes, enabled free viewing or downloading journal's website content, created portals to view specific content related to COVID-19, and provided free access to all pandemic-related articles.

The aim of the present study is to analyze the extent to which the publication process of scientific papers in the field of otolaryngology-head and neck surgery (ORL-HNS) related and unrelated to the pandemic is being expedited in the COVID-19 era.

## Materials and Methods

### Ethical Consideration

The present study did not require approval from the institutional review board nor that of the ethical committee according to local law because it does not use individualized patient data.

### Data Collection and Analysis

We conducted an online search to analyze the processing times of original articles published in selected ORL-HNS peer-reviewed journals. We divided the times into those from submission to acceptance (S-A), from acceptance to publication (A-P), and from submission to publication (S-P). The search was limited to ORL-HNS manuscripts that appeared during the COVID-19 era compared with those that were published during the pre-COVID-19 era. We also compared the S-A, A-P, and S-P times between papers submitted during the COVID-19 era that were and were not related to COVID-19.

Five arbitrarily selected ORL-HNS journals in which the dates of submission, acceptance, and publication were available were included in the study. We chose the first 5 articles of each issue according to 5 otolaryngology subdisciplines (rhinology and paranasal sinuses, laryngology, otology/neurotology, comprehensive [general] otolaryngology, and head and neck) from February 2019 until March 2021. We recorded the 3 relevant dates for each included article.

### Statistical Methods

Categorical variables were summarized as frequencies and percentages. Continuous variables were evaluated for normal distribution with histograms and Q-Q plots and reported as median and interquartile range (IQR) since none was normally distributed. The Pearson correlation coefficient was used to evaluate the correlation between the 3 time intervals, and a violin plot was applied for demonstration. A paired samples t-test was applied to evaluate the absolute difference between the time intervals. The Kruskal-Wallis test and the Mann-Whitney test were used to compare the differences between the time intervals in the pre-COVID-19 and COVID-19 eras. All statistical tests were 2-sided, and *p* < 0.05 was considered significant. All statistical analyses were performed by IBM SPSS Statistics for Windows version 25 (IBM Corp., Armonk, NY, USA).

## Results

A total of 487 articles were included from 5 arbitrarily selected ORL-HNS journals: *Otolaryngology-Head and Neck Surgery* ( *n* = 122; 25.1%), *Laryngoscope Investigating Otolaryngology* ( *n* = 56; 11.5%), *European Archives of Otorhinolaryngology* ( *n* = 119; 24.4%), *Auris Nasus Larynx* ( *n* = 70; 14.4%), and *Acta Otolaryngologica* ( *n* = 120; 24.6%). The distribution of papers between the various ORL-HNS disciplines was as follows: comprehensive (general) otolaryngology ( *n* = 96; 19.7%), head and neck ( *n* = 88; 18.1%), otology/neurotology ( *n* = 131; 26.9%), rhinology and paranasal sinuses ( *n* = 94; 19.3%), and laryngology ( *n* = 78; 16.0%) ( Tables 1 and 2 ).

**Table 1:** Description of the 5 Leading ORL-HNS Journals in Our Analyses

Journal	Country of Origin	Number of Issues Per Year	Founded	Official Journal Society
Otolaryngology Head and Neck Surgery	USA	12	1978	AAO-HNS, the American Academy of Otolaryngology-Head and Neck Surgery Foundation
Laryngoscope Investigative Otolaryngology	USA	6	2016	The American Laryngological, Rhinological and Otological Society, Inc.
European Archives of Oto-Rhino-Laryngology	Germany	12	1864	Confederation of European Oto-Rhino-Laryngology Head and Neck Surgery
Auris Nasus Larynx	Japan	6	1973	The Oto-Rhino-Laryngological Society of Japan, Inc.
Acta Otolaryngologica		12	1918	

**Table 2:** Distribution of Enrolled Papers According to Journal and Discipline

			Journal					Total
			Otolaryngology, Head and Neck Surgery	Auris Nasus Lartynx	Acta Otolaryngologica	European Archives Otolaryngology	Laryngoscope Investigative Otolaryngology	
Discipline	Rhinology and para-nasal sinuses	Count	23	16	20	23	12	94
		% within Discipline	24.5%	17.0%	21.3%	24.5%	12.8%	100.0%
		% within Journal	18.9%	22.9%	16.7%	19.3%	21.4%	19.3%
		% of Total	4.7%	3.3%	4.1%	4.7%	2.5%	19.3%
	Laryngology	Count	20	12	15	22	9	78
		% within Discipline	25.6%	15.4%	19.2%	28.2%	11.5%	100.0%
		% within Journal	16.4%	17.1%	12.5%	18.5%	16.1%	16.0%
		% of Total	4.1%	2.5%	3.1%	4.5%	1.8%	16.0%
	Otology/ Neurotology	Count	21	16	60	23	11	131
		% within Discipline	16.0%	12.2%	45.8%	17.6%	8.4%	100.0%
		% within Journal	17.2%	22.9%	50.0%	19.3%	19.6%	26.9%
		% of Total	4.3%	3.3%	12.3%	4.7%	2.3%	26.9%
	Comprehensive (general) ORL	Count	35	11	11	27	12	96
		% within Discipline	36.5%	11.5%	11.5%	28.1%	12.5%	100.0%
		% within Journal	28.7%	15.7%	9.2%	22.7%	21.4%	19.7%
		% of Total	7.2%	2.3%	2.3%	5.5%	2.5%	19.7%
	Head and Neck	Count	23	15	14	24	12	88
		% within Discipline	26.1%	17.0%	15.9%	27.3%	13.6%	100.0%
		% within Journal	18.9%	21.4%	11.7%	20.2%	21.4%	18.1%
		% of Total	4.7%	3.1%	2.9%	4.9%	2.5%	18.1%
Total		Count	122	70	120	119	56	487
		% within Discipline	25.1%	14.4%	24.6%	24.4%	11.5%	100.0%
		% within Journal	100.0%	100.0%	100.0%	100.0%	100.0%	100.0%
		% of Total	25.1%	14.4%	24.6%	24.4%	11.5%	100.0%

Almost one-half ( *n* = 236; 48.5%) of the included articles were published during the pre-COVID-19 era, and 251 (51.5%) articles were published during the COVID-19 era, of which 180 (37%) were unrelated to COVID-19 and 71 (14.5%) were related to COVID-19.

**Fig. 1:**
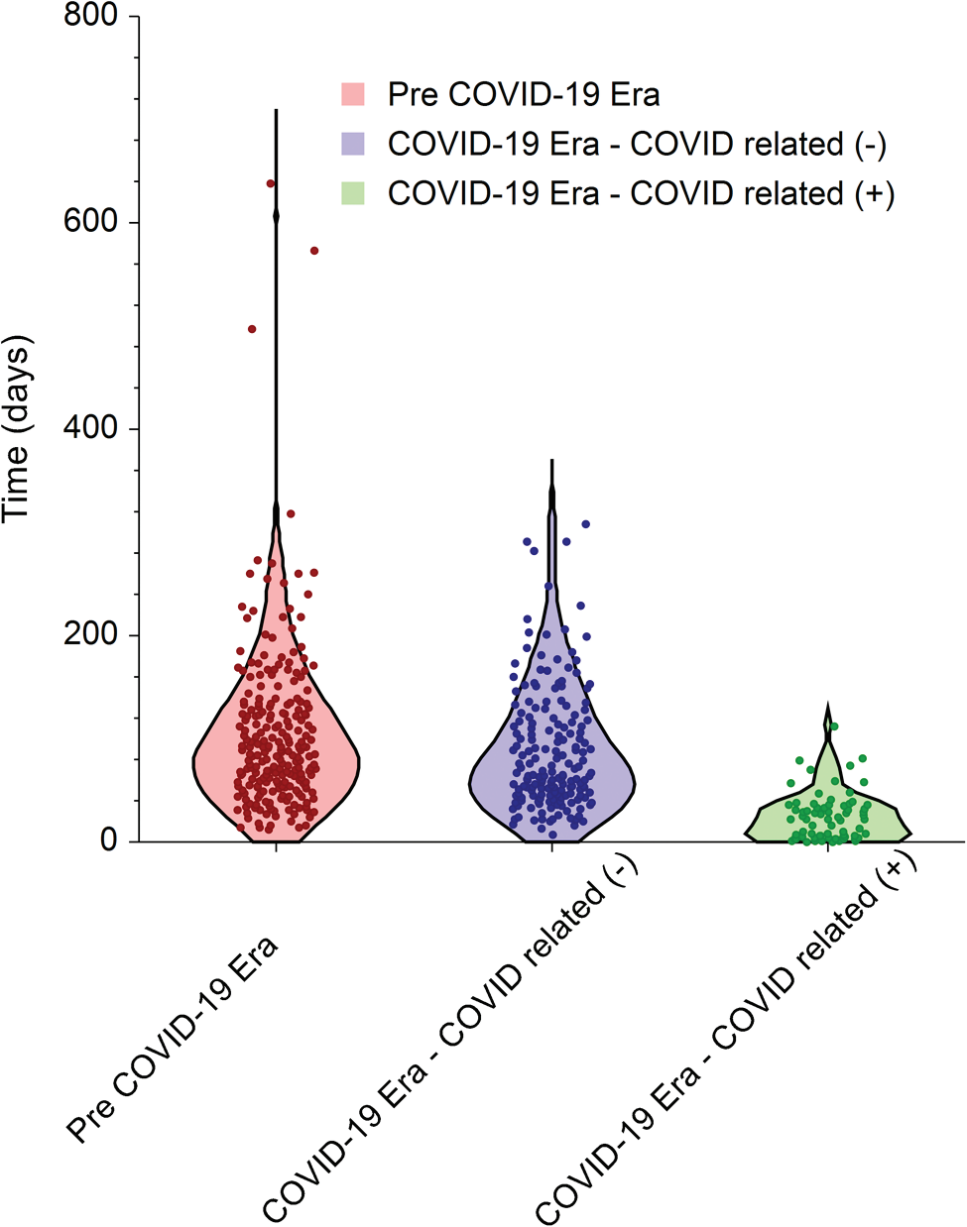
Violin plot of S-A duration for all included articles. Each dot represents an enrolled article. The width of the plot represents the probability density.

**Fig. 2:**
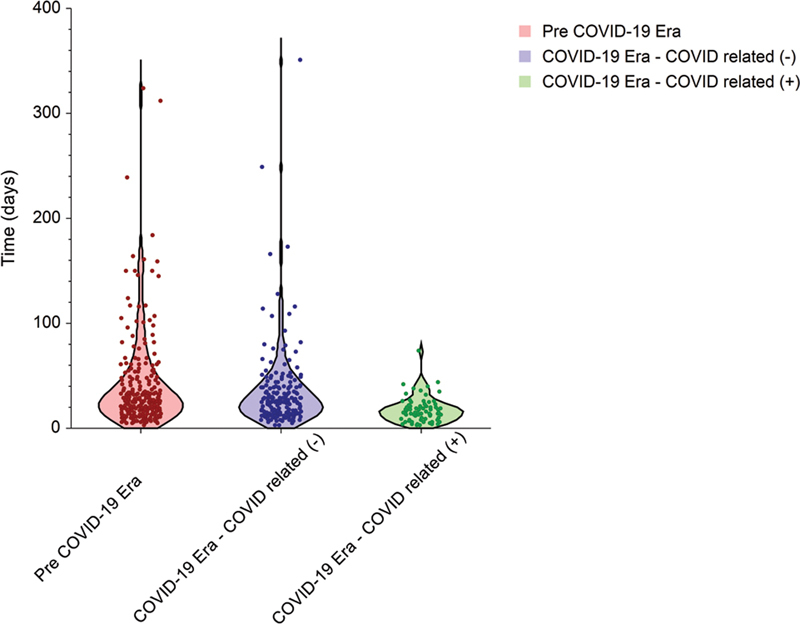
Violin plot of accepted-publication (A-P) duration for all articles included. Each dot represents an enrolled article. The width of the plot represents the probability density.

**Fig. 3:**
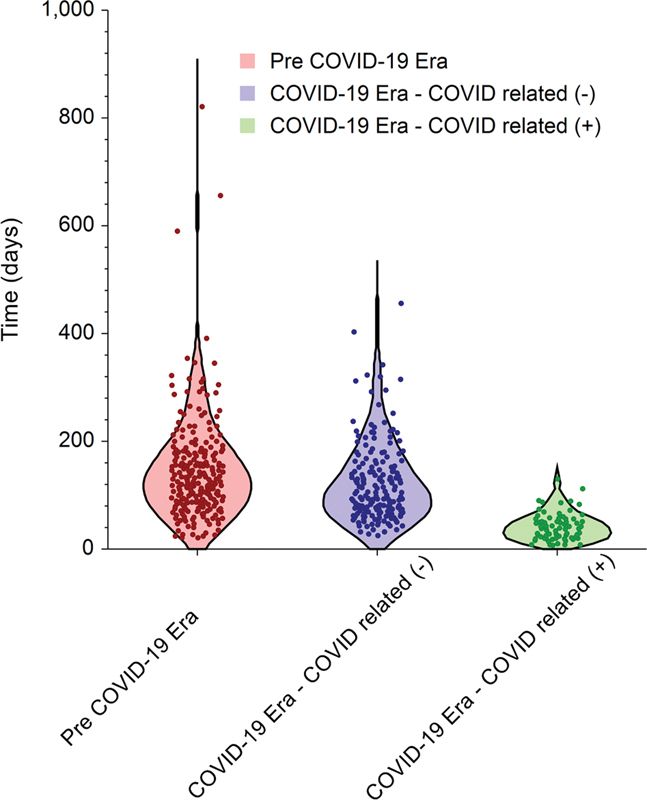
Violin plot of submission-publication (S-P) duration for all articles included. Each dot represents an enrolled article. The width of the plot represents the probability density.

The overall median (IQR) S-A duration was 69 (range 39 to 118) days, and the overall median (IQR) A-P duration was 25 (14 to 39) days, resulting in an overall median (IQR) S-P duration of 104 (63 to 159) days. Stratification of the durations according to submission time showed that the median (IQR) S-A, A-P, and S-P durations in the pre-COVID-19 era were 88 (55 to 133), 27 (17 to 48), and 130 (86 to 180) days, respectively. In contrast, the median (IQR) S-A, A-P, and S-P durations during the COVID-19 era were 68 (45 to 120), 26 (15 to 40), and 105 (72 to 157) days for papers unrelated to COVID-19, while papers related to COVID-19 were processed at durations of 22 (5 to 36), 15 (9 to 22), and 39 (21 to 56) days, respectively. The shorter processing durations of the COVID-19-related papers compared with the unrelated papers reached a level of statistical significance ( *p* < 0.001 for all 3 durations). The same applied to the ORL-HNS papers submitted before the COVID-19 era compared with the COVID-19-related ORL-HNS papers ( *p* < 0.001 for all 3 durations). Interestingly, papers that were unrelated to COVID-19 that were processed during the COVID-19 era were also processed more rapidly (S-A and S-P durations) than the papers that had been submitted during the pre-COVID-19 era ( Table 3 , Figs. 1 , 2 , 3 ).

**Table 3:** Median (IQR), of Processing Durations (days) for Included Articles

Era	Median (IQR), of processing durations (days)								
	S-A	p Value*		A-P	p Value*		S-P	p Value*	
Pre-COVID 19 Era	89 (56-133)	=.014 <.001	<.001	27 (17-48)	<.001	<.001	130 (86-180)	=.004 <.001	<.001
COVID 19 Era, unrelated	68 (45-120)			26 (15-40)			105 (72-158)		
COVID 19 Era, related	22 (5-36)			15 (19-22)			39 (21-56)		

* Only p Values <0.05 are shown.

Additionally, stratification of processing durations was performed as a function of the journal. The S-A duration of COVID-19-related articles was significantly shorter compared with that of papers submitted in the pre-COVID-19 era and with papers submitted in the COVID-19 era but that were unrelated to COVID-19 (a median of 6 to 34 days compared with 65 to 125 and 46 to 127, respectively) in all selected journals. The A-P duration of papers related to COVID-19 was significantly shorter than that of papers unrelated to COVID-19 in 4/5 journals (median 6 to 19 days compared with 11 to 51 days). This shorter A-P duration reflects more rapid editing, proofreading, and graphical designing of the editorial and production teams of the individual journals. The resultant S-P duration of papers related to COVID-19 compared with other papers was significantly shorter in 4 out of 5 journals (median 8 to 51 days compared with 70 to 184 days) ( *Table 4* ).

**Table 4:** Median (IQR), of Processing Durations (days) per Individual Journal

Journal	Era	Median (IQR), of processing durations (days)								
		S-A	p Value*		A-P	p Value*		S-P	p Value*	
Otolaryngology, Head and Neck Surgery	Pre-COVID 19 Era	125 (92-167)	<.001	<.001	28 (25-49)	=.034 <.001	<.001	159 (123-217)	<.001	<.001
	COVID 19 Era unrelated	127 (68-183)			41 (29-51)			184 (137-226)		
	COVID 19 Era, Related	6 (2-28)			19 (15-24)			8 (20-48		
Auris Nasus Larynx	Pre-COVID 19 Era	124 (73-174)	<.001	<.001	20 (15-26)	=.001	<.001	153 (102-199)	<.001	<.001
	COVID 19 Era unrelated	111 (74-154)			17 (14-25)			136 (95-177)		
	COVID 19 Era, Related	6 (3-19)			6 (4-14)			16 (8-27)		
Acta Otolaryngologica	Pre-COVID 19 Era	71 (51-90)	<.001 =.011	=.001	38 (29-89)	=.002		118 (89-163)	<.001	=.001
	COVID 19 Era unrelated	46 (38-63)			29 (25-39)			79 (64-107)		
	COVID 19 Era, Related	34 (22-37)			37 (21-52)			62 (50-80)		
European Archives Otolaryngology	Pre-COVID 19 Era	65 (34-111)	=.003	=.006	11 (7-17)	=.004	=.001	78 (47-129)	=.002	=.004
	COVID 19 Era unrelated	62 (36-109)			12 (6-16)			70 (50-126)		
	COVID 19 Era, Related	29 (22-58)			6 (4-9)			37 (29-74)		
Laryngoscope Investigative Otolaryngology	Pre-COVID 19 Era	105 58-135)	=.002	<.001	48 (28-65)	<.001	<.001	149 (90-213)	<.001	<.001
	COVID 19 Era unrelated	88 (53-128)			51 (33-73)			128 (97-217)		
	COVID 19 Era, Related	33 (15-56)			15 (13-22)			51 (41-75)		

* Only p Values <0.05 are shown.

Stratification according to ORL-HNS disciplines demonstrated variability in processing times. The most impressive reduction in S-A times was documented in the laryngology disciplines, where papers submitted in the pre-COVID-19 era had been processed significantly slower than papers submitted during the COVID-19 era, independently of any relation to COVID-19 (median 106 days compared with 61 days for unrelated papers and 34 days for papers related to COVID-19, respectively). The results in the S-P duration in the otology/neurotology discipline were similar (median 123 days compared with 104 days for unrelated papers and 46 days for papers related to COVID-19, respectively). *Table 5* lists the processing times for the individual disciplines.

**Table 5:** Median (IQR), of Processing Durations (days) per Individual Discipline

Disipline	Era	Median (IQR), of processing durations (days)								
		S-A	p Value*		A-P	p Value*		S-P	p Value*	
Rhinology and para-nasal sinuses	Pre-COVID 19 Era	93 (55-140)	<.001	<.001	26 (16-49)			134 (90-188)	<.001	<.001
	COVID 19 Era, unrelated	88 (54-129)			25 (14-39)			116 (73-166)		
	COVID 19 Era, Related	28 (8-33)			19 (12-33)			50 (24-66)		
Laryngology	Pre-COVID 19 Era	106 (65-173)	=.028 =.006	=.002	25 (12-47)	=.009		146 (77-218)	=.002	
	COVID 19 Era, unrelated	61 (38-105)			27 (15-40)			92 (62-177)		
	COVID 19 Era, Related	34 (4-38)			14 (9-19)			46 (16-56)		
Otology/ Neurotology	Pre-COVID 19 Era	79 (53-113)	=.016	=.005	33 (21-56)		=.021	123 (92-171)	=.048 =.004	=.001
	COVID 19 Era, unrelated	67 (45-126)			26 (17-44)			104 (75-144)		
	COVID 19 Era, Related	26 (11-64)			18 (7-28)			46 (34-85)		
Comprehensive (general) ORL	Pre-COVID 19 Era	70 (49-131)	<.001	<.001	25 (14-34)		=.004	110 (74-171)	<.001	<.001
	COVID 19 Era, unrelated	55 (33-90)			17 (14-41)			78 (47-154)		
	COVID 19 Era, Related	13 (3-36)			16 (9-23)			35 (20-51)		
Head and Neck	Pre-COVID 19 Era	99 (64-134)	=.002	=.002	26 (12-37)			131 (99-170)	=.001	=.003
	COVID 19 Era, unrelated	79 (51-130)			27 (12-40)			121 (77-178)		
	COVID 19 Era, Related	25 (9-29)			8 (5-17)			33 (13-46)		

Only p Values <0.05 are shown.

## Discussion

It was our impression that our manuscripts that were related to the COVID-19 pandemic – and those unrelated as well – were being processed by the target journals more quickly than usual. Our colleagues in other fields also shared this observation. We were therefore curious to analyze the impact of the COVID-19 pandemic on journal processing times before publication in our own field of ORL-HNS.

According to our analysis, article processing times for the arbitrarily selected ORL-HNS journals prior to the COVID-19 era were indeed substantially slower compared to the processing times during the COVID-19 era, and COVID-19-related papers were generally processed more quickly than non-COVID-19-related papers. Our analysis results are in accordance with a previously published analysis. 3

It has become standard practice for some journals to suggest an option for fast-track publication not necessarily in regard to PHEICs. In the presence of the swift spread of the COVID-19 pandemic and the appearance of new strains of the virus world-wide, high flow of shared published information and evidence enables the scientific and medical community as well as governments and societies to cope better with the disease and the PHEIC.

The substantial world-wide influence of the current COVID-19 pandemic inarguably necessitates expediting research regarding the new virus and publication of the research findings, especially findings that could impact on the virus spreading and the disease management. However, Ioannidis suggested that the exceptional publication pace and volume of research regarding COVID-19 might be concerning in the aspect of maintaining high standard evidence base that is reliable and credible, without spreading wrong information that might be misleading and destructive. 4 Thus, now, more than ever, it is essential to maintain the high standard supervision of the journals by the editors and the reviewers in order to preserve the quality and integrity of the published information and evidence, to withhold it from being misleading and confusing, resulting in damageable public and scientific consequences. Dealing with the COVID-19 new challenge, in which much is still unknown, the search for new information published quickly to the scientific and medical community will probably continue in the near future.

Scientific publishing is naturally evolving in the presence of new challenges and demands. The current challenge is to enable fast publication of contributing research regarding the COVID-19 PHEIC, without damaging the quality, credibility, and reliability of the evidence published and the scientific publishing process.

One of the lessons from the current COVID-19 pandemic is that scientific evidence and information should be shared and transferred from researchers to the healthcare providers who can implement them as rapidly as possible. This can occur at different rates depending upon the scientific review process, the timeliness of administrative approval, and the will of individuals to share their information with the broad scientific community. It is clear that the medical community and thus the public may benefit by shortening the time interval from discovery to application.

The increased rate and volume of scientific research and the swift dissemination of information through peer review process and publication during the current pandemic have been exceptional. Processes that have usually taken months have been reduced to weeks or even days. Maintaining the favorable changes in the way medical and scientific journals deal with the epidemiological and information on the COVID-19 crisis during the post-COVID-19 era will be of profound benefit. Apparently, the review process can be accelerated, and publishers can enable larger volume of preprint articles. It seems that journal editorial boards could preserve those expedited publishing rates by publishing free of charge open access articles that provide significant and pivotal evidence and information for international public health.

We would be remiss by not acknowledging our ignorance on the cost incurred by medical publishers to accomplish these feats, be it financial or extraordinary efforts on the part of the publications' staff members or both. We can only hope that their efforts and motivation will be sustained when the pandemic is finally over.

## Conclusions

Processing times of the included papers were significantly shorter in most of the selected ORL-HNS journals and in most of the ORL-HNS disciplines during the COVID-19 era compared with the pre-COVID-19 era. COVID-19-related papers were also processed more rapidly than non-COVID-19-related papers during the COVID-19 era. Publishers should be congratulated for their dedication to expeditiously disseminate vital data to the medical community and to the public at large. It is hoped that this momentum will be maintained in a COVID-19-free future.
